# CILP2 is a potential biomarker for the prediction and therapeutic target of peritoneal metastases in colorectal cancer

**DOI:** 10.1038/s41598-024-63366-4

**Published:** 2024-05-31

**Authors:** Ye Jin Ha, Seong-Hwan Park, Ka Hee Tak, Jong Lyul Lee, Chan Wook Kim, Jeong-Hwan Kim, Seon-Young Kim, Seon-Kyu Kim, Yong Sik Yoon

**Affiliations:** 1 Asan Institute for Life Sciences, Asan Medical Center, University of Ulsan College of Medicine, Seoul, 05505 Korea; 2 Aging Convergence Research Center, Korea Research Institute of Bioscience and Biotechnology (KRIBB), Daejeon, 34141 Korea; 3 Department of Bioscience, University of Science and Technology, Daejeon, 34113 Korea; 4 Division of Colon and Rectal Surgery, Department of Surgery, Asan Medical Center, University of Ulsan College of Medicine, Seoul, 05505 Korea; 5 Personalized Genomic Medicine Research Center, KRIBB, Daejeon, 34141 Korea; 6 Korea Bioinformation Center, KRIBB, Daejeon, 34141 Korea

**Keywords:** Cancer, Gastroenterology

## Abstract

Peritoneal metastases (PM) in colorectal cancer (CRC) is associated with a dismal prognosis. Identifying and exploiting new biomarkers, signatures, and molecular targets for personalised interventions in the treatment of PM in CRC is imperative. We conducted transcriptomic profiling using RNA-seq data generated from the primary tissues of 19 CRC patients with PM. Using our dataset established in a previous study, we identified 1422 differentially expressed genes compared to non-metastatic CRC. The profiling demonstrated no differential expression in liver and lung metastatic CRC. We selected 12 genes based on stringent criteria and evaluated their expression patterns in a validation cohort of 32 PM patients and 84 without PM using real-time reverse transcription-polymerase chain reaction. We selected cartilage intermediate layer protein 2 (CILP2) because of high mRNA expression in PM patients in our validation cohort and its association with a poor prognosis in The Cancer Genome Atlas. Kaplan–Meier survival analysis in our validation cohort demonstrated that CRC patients with high CILP2 expression had significantly poor survival outcomes. Knockdown of CILP2 significantly reduced the proliferation, colony-forming ability, invasiveness, and migratory capacity and downregulated the expression of molecules related to epithelial-mesenchymal transition in HCT116 cells. In an in vivo peritoneal dissemination mouse knockdown of CILP2 also inhibited CRC growth. Therefore, CILP2 is a promising biomarker for the prediction and treatment of PM in CRC.

## Introduction

Colorectal cancer (CRC) is the third most common cancer and the second leading cause of cancer-related mortality worldwide^1,2^. Metastases in the liver, peritoneal cavity, and lungs are the primary contributors to mortality in CRC patients. Notably, peritoneal metastases (PM) in CRC are associated with the worst prognosis^3–6^. The incidence of PM in CRC reportedly ranges from 17 to 40% of concurrent primary cancer patients and 44–50% of recurrent cases^7–9^. Despite the formulation of consensus guidelines for PM treatment from CRC^10^, PM presents a particularly aggressive prognosis, resulting in poor overall survival compared to other metastatic sites^5^. Early detection of PM is challenging, primarily due to the absence of typical symptoms and the limited accuracy of current imaging modalities^11–13^. Surgical removal of PM is often complicated as the cancer cells tend to spread extensively across the peritoneum^14,15^, and conventional anticancer or immunotherapeutic agents have displayed limited efficacy against the aforementioned metastases^16,17^.

The principal processes underlying CRC liver or lung metastases occur through the lymphatic system and blood vessels. In contrast, direct seeding into the peritoneal cavity is considered the most important pathway for PM^1,18^. The primary tumour cells invade the intestinal wall, leak into the abdominal cavity, resist apoptosis, migrate, and attach to the peritoneal surface, thus metastasising to the peritoneum. However, these mechanisms have not been elucidated, and research on the molecular characteristics of PM in CRC is scant. Therefore, understanding the molecular characteristics of PM in CRC may be necessary for early diagnosis and may help to improve the management of such patients.

In this study, we conducted RNA-seq analysis to identify candidate molecules associated with PM in CRC. To validate gene expression patterns, we used real-time reverse transcription polymerase chain reaction (RT-PCR) in our validation cohort. Furthermore, we validated the relationship between overall survival and gene expressions in The Cancer Genome Atlas (TCGA) and our validation cohorts. Finally, we conducted in vitro and in vivo experiments to assess the diagnostic and therapeutic significance of the candidate gene in the CRC cell line and mouse xenograft model.

## Materials and methods

### Patient enrolment and sample acquisition

A total of 113 CRC patients were included in the RNA-seq analysis. Transcriptomic profiles of non (n = 62), liver (n = 27), or lung (n = 5) metastases in CRC tumour tissues were obtained from our previously reported RNA-seq data (GSE50760 and GSE107422)^19^. Additionally, 19 primary tumour tissues from CRC patients with PM were collected at the Asan Medical Center (Seoul, Korea) for RNA-seq in this study. No patients underwent preoperative treatment, peritonectomy, or hyperthermic intraperitoneal chemotherapy. For the validation assay, we collected another set of primary tumour tissues from an independent validation cohort (n = 116), comprising PM (n = 32) and non-PM (n = 84) CRC patients. Table S1 presents the clinicopathological features of the RNA-seq and validation cohorts.

### RNA-seq and data processing

RNA was purified from the primary CRC tissues using the RNeasy Mini Kit (Qiagen, Inc.). Bioanalyzer (Agilent Technologies, Inc.) was used to measure the concentrations and purities of the RNA preparations. After total RNA isolation, a sequencing library was prepared using the TruSeq RNA sample preparation kit v2 (Illumina, Inc.) in accordance with the manufacturer's instructions. The mRNA was purified from total RNA extracts using poly-T oligo-attached magnetic beads, fragmented, and converted into cDNA. Sequencing was performed in paired-end reads (2 × 150 bp) using the Hiseq-4000 sequencing system (Illumina, Inc.). The reference genome index was built using SAMtools (ver. 0.1.18), and samples were quantified using Kallisto (ver. 0.43.0). The sequencing reads were mapped to the human reference genome, GRCh38. Table S2 presents the sequencing coverage and quality statistics of each sample. We employed the EdgeR package with a negative binomial model to assess the significance of gene expression differences between sample subgroups from count data^20^.

### Real-time reverse transcription–polymerase chain reaction

The cDNA samples were synthesised from total RNA preparations using random primers and SuperScript II RT (Thermo Fisher Scientific, Inc.). Real-time RT-PCR was performed on these samples using a Roche LightCycler 96 with SYBR Green I Master Mix (Roche Life Science). The primers used to amplify target genes are listed in Table S3. The glyceraldehyde 3-phosphate dehydrogenase gene was used as an internal control.

### Cell culture and gene transfection

CRC cell lines (HCT15, HCT116, HCT116-Luc, LoVo, RKO, and SW480) were obtained from the American Type Culture Collection. All cell lines were confirmed to be free of mycoplasma and authenticated using purified DNAs on a 3130 × 1 genetic analyser with the GeneMapper software ver. 5 (Cosmogenetech, Inc). These cells were cultured in RPMI-1640 medium (Invitrogen, Thermo Fisher Scientific, Inc.) supplemented with 10% (v/v) fetal bovine serum and 1% (w/v) penicillin and streptomycin. For gene knockdown, a small interfering RNA (siRNA) directed against the CILP2 gene (Bioneer, Inc.) was transfected into cells using the RNAiMax transfection reagent (Thermo Fisher Scientific, Inc.). Negative control siRNA (siNC) was acquired from Bioneer. The CILP2 short hairpin RNA (shRNA) plasmid kit was purchased from OriGene. HCT116-Luc cells stably transfected with shRNA plasmid were used to establish the PM mouse model.

### Western blotting

For Western blotting, the protein concentrations of extracts from the cultured CRC cells were first quantified using Bradford solution (Bio-Rad Laboratories, Inc.). The proteins were subsequently resolved by SDS-PAGE and then transferred to the polyvinylidene difluoride membrane (Merck Millipore Ltd). The membranes were consecutively incubated with primary and secondary antibodies. Specific complexes were detected using the SuperSignal West Pico kit (Thermo Fisher Scientific, Inc.). The following antibodies were used: anti-CILP2 from Santa Cruz, anti-E-Cadherin, anti-N-Cadherin, anti-matrix metalloproteinase (MMP) 9, anti-MMP2 from Abcam, anti-Claudin-1 from Cell Signaling Technology, and anti-β-Actin, anti-mouse IgG, and anti-rabbit IgG from Bethyl Laboratories.

### Proliferation, colony formation, migration, and invasion assays

We seeded cells onto 96-well plates to assess proliferation. We measured daily cell fold changes over 5 days using a cell counting kit-8 cell proliferation assay kit (DOJINDO Laboratories) on a Tecan microtiter plate reader set at 450 nm absorbance. For the colony-forming assay, transfected cells were seeded into 6-well plates (400 cells/well) and cultured at 37 °C for 7 days. After colonies had formed, they were fixed with 100% methanol, stained with 0.2% crystal violet, and counted using a GelCount™ system (Oxford Optronix Ltd.). For invasion and migration assays, the cells (1 × 10^5^ cells/well) were seeded in the upper chambers of 24-well culture plates on Biocoat™ Matrigel invasion chambers and Transwell chambers (Corning, Inc.), respectively. Additionally, a 3T3-fibroblast-conditioned medium was placed in the lower chamber as a chemoattractant. After incubation at 37 °C for 24 h, the cells on the lower surface of the membrane were stained with 0.2% crystal violet and counted in four different fields under a light microscope.

### In vivo peritoneal metastases model of colorectal cancer

Five-week-old male BALB/c-nu mice were purchased from Orient Bio (Orient Bio Inc.) to generate a PM of the CRC model. The condition and body weights of the mice were assessed twice weekly. We injected 5 × 10^6^ HCT116-Luc cells, resuspended in 100 μL of RPMI culture media mixed with 100 μL Matrigel (Corning, Inc.), into the mice’s peritoneal cavity. The Lumina in vivo Imaging System (PerkinElmer, Inc.) was used to measure the PM volumes non-invasively. D-luciferin (150 mg/kg) (BioVision, Inc.) was injected intraperitoneally at 1, 2, 3, and 4 weeks after cell implantation, and luciferase activity was detected for the first 10 min after the injection. Living Image® version 4.7.2 software (Xenogen, Inc.) was used to acquire and analyse the data. The mice were sacrificed after 4 weeks, and the tumours and ascites in the peritoneal cavity were isolated.

### Statistical analysis

Differential mRNA expression was compared between the two groups using the Mann–Whitney *U*-test. To calculate the best cut-off for expression of each gene, receiver operating characteristics (ROC) analysis was performed, and the optimal cut-off value was determined as the expression level with the highest sensitivity and specificity. Overall survival (OS) was compared using the Kaplan–Meier method with the log-rank test. The χ^2^ test and *t*-test were used to compare patient groups. The differences between the experimental and control groups were compared using a *t*-test. Data are expressed as the mean ± standard deviation. All statistical comparisons were performed using SPSS21 (IBM Corp.).

### Ethical approval and consent to participate

The study was conducted in accordance with the Declaration of Helsinki and was approved by the Institutional Review Board of the Asan Medical Center (Approval No.: 2020-0287). Written informed consent was obtained from all patients and/or their legal guardians. All animal experimental protocols were approved by the Institutional Animal Care and Use Committee of the Asan Institute for Life Sciences, Seoul, Korea (IACUC2021-02-337). All methods are reported in accordance with ARRIVE guidelines. All methods were carried out in accordance with relevant guidelines and regulations.

## Results

### Differential gene expression among the primary CRC samples

**Figure 1:**
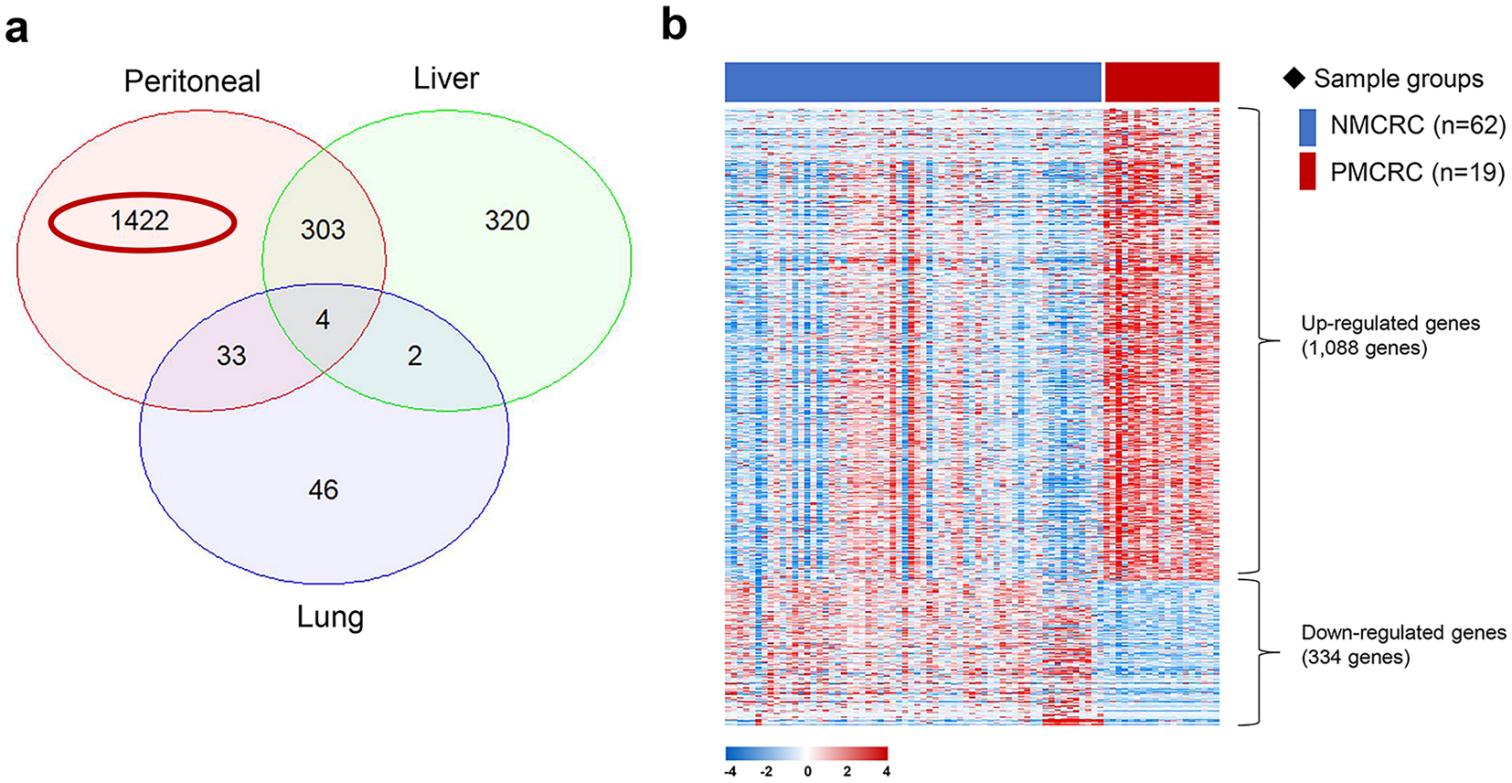
Transcriptomic profiling analysis. (**a**) Venn diagram of differentially expressed genes (DEG) in primary liver, lung, and peritoneal metastatic colorectal cancer (PMCRC) in comparison to primary tumours in non-metastatic CRC (NMCRC). (**b**) Heatmap including 1,422 DEG between the PMCRC and NMCRC groups.

We performed transcriptomic profiling of CRC samples to identify genes that were expressed in different patterns in the PM of CRC. We filtered a panel of 1,422 genes according to three criteria: (1) significant difference (*P* > 0.001) between PM in CRC (PMCRC) and non-metastatic CRC (NMCRC); (2) no significant difference (*P* > 0.001) between liver metastases and NMCRC; and (3) no significant difference (*P* > 0.001) between lung metastases and NMCRC (Table S4 and Fig. 1a). The differentially expressed genes (DEG) in the PMCRC included 1,088 genes that were upregulated compared with NMCRC and 334 that were downregulated (Fig. 1b). These candidates were further narrowed to 12 genes for validation by the following criteria: Bonferroni *P* < 0.001, logFC (logarithmic fold change of the gene expression) > 3, logCPM (logarithmic counts per million reads) > 1 (Table S3).

### Validation of the differential expression of genes by real-time RT-PCR

**Figure 2:**
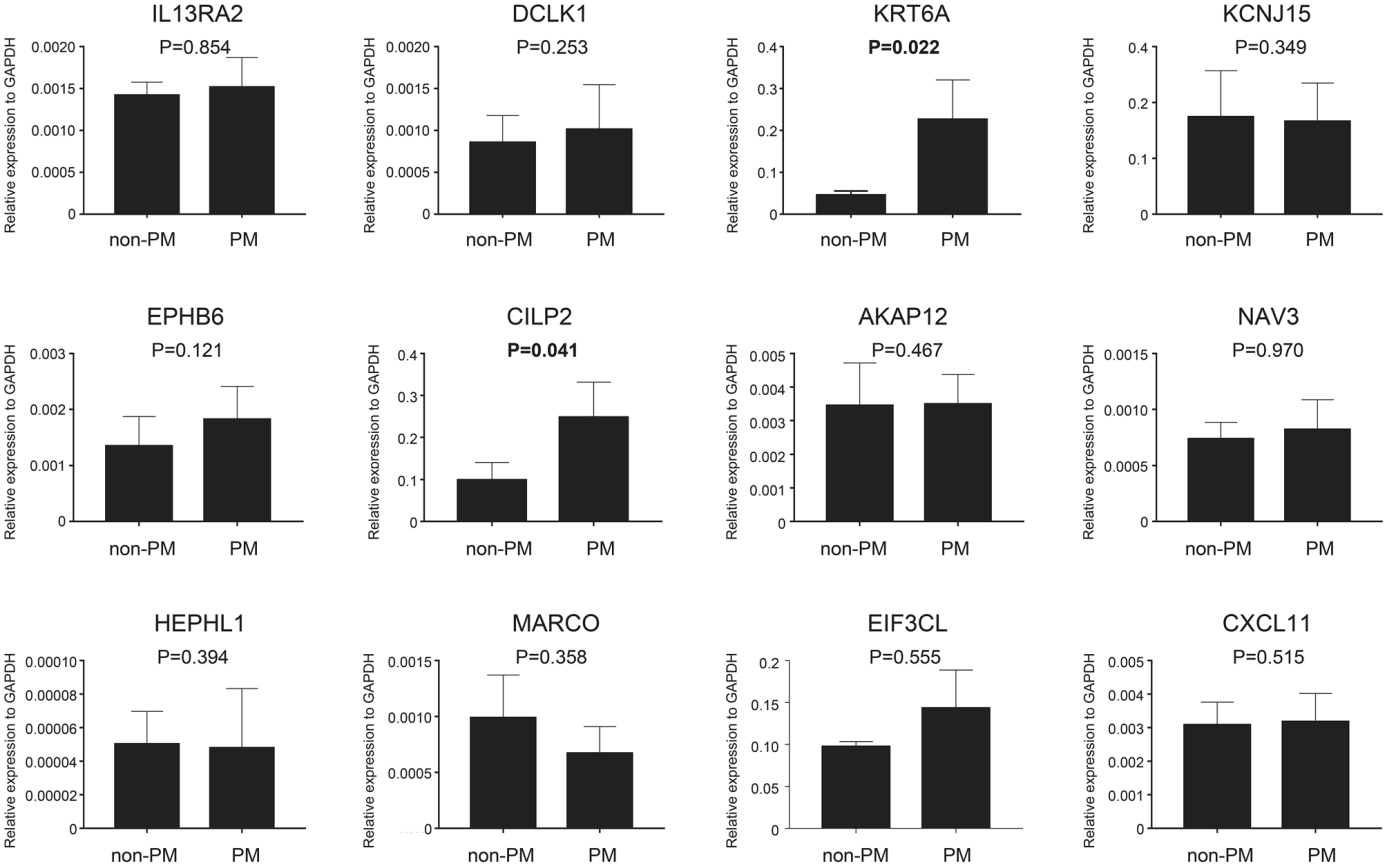
The messenger RNA expression levels of the differentially expressed genes in a validation cohort by real-time reverse transcription–polymerase chain reaction (RT-PCR) in 116 CRC cases with non-PM (n = 84) and PM (n = 32) analysed by real-time RT-PCR.

To validate the differential gene expression of 12 genes between the PM and non-PM tissues, the mRNA levels were determined using real-time RT-PCR. CILP2 and KRT6A expression levels were significantly higher in patients with PM compared with non-PM patients. The other ten genes demonstrated no significant differences in mRNA expression in the validation cohort (Fig. 2).

### High expression of CILP2 predicts a poor prognosis for patients with CRC

**Figure 3:**
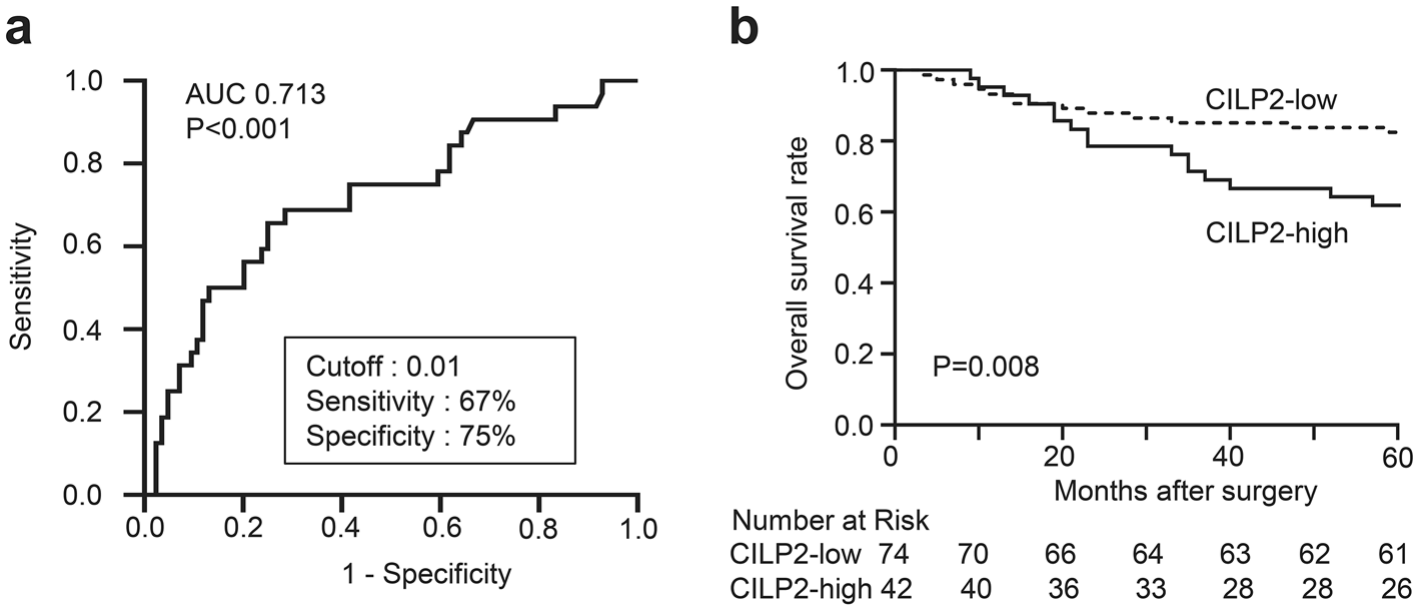
The prognostic value of CILP2 in the validation cohort of CRC (n = 116). (**a**) Receiver-operating characteristic curve analysis of the CILP2 expression values is conducted to predict PM. (**b**) Kaplan–Meier survival curves are generated in accordance with their CILP2 expression classification.

We assessed the relationship between CILP2 or KRT6A expression and CRC prognosis using data from the TCGA-CRC datasets available in the human protein atlas. TCGA-CRC displayed that the group with a high expression of CILP2 was associated with a reduced survival rate in patients with CRC (https://www.proteinatlas.org/ENSG00000160161-CILP2/pathology/colorectal+cancer, Fig. S1). Conversely, the group with high KRT6A expression displayed a favourable prognosis (https://www.proteinatlas.org/ENSG00000205420-KRT6A/pathology/colorectal+cancer, data not displayed). The relationship between CILP2 expression and the prognosis of CRC was further evaluated in our validation cohort. Furthermore, ROC curve analysis was conducted to evaluate the sensitivity and specificity of CILP2 for diagnosing PMCRC. The area under the ROC curve of CILP2 mRNA expression was 0.713, thus demonstrating a high sensitivity and specificity for PMCRC diagnosis (Fig. 3a). Kaplan–Meier analysis was also performed using the validation cohort. Patients with a high expression of CILP2 had a significantly poor OS outcome (Fig. 3b), which is consistent with the findings in the TCGA cohort. When we used this cut-off value to designate high and low CILP2 expression groups, high CILP2 mRNA levels in the primary CRC tissues were associated with PM (high CILP2: 50% vs. low CILP2: 15%, *P* = 0.00008 (Table 1). High CILP2 mRNA levels also demonstrated an association with a poor level of differentiation and tumour stage. Analysis of TCGA data revealed a significantly poor OS in patients with CRC and high CILP2 expression. Based on these results of prognostic analysis, CILP2 was selected for biological functional analysis.

**Table 1:** Association between the CILP2 mRNA expression and clinicopathological characteristics of the patients included in the validation cohort.

Variables	High CILP2 (n = 42)	Low CILP2 (n = 74)	*P* value*
Age (years), mean ± SD	59.50 ± 10.49	59.26 ± 11.93	0.91
Sex			0.44
Male	23 (55%)	46 (62%)	
Female	19 (45%)	28 (38%)	
Preoperative CEA, ng/mL			0.15
≤ 5	24 (57%)	53 (72%)	
> 5	18 (43%)	21 (28%)	
Peritoneal metastases			**0.00008**
Negative	21 (50%)	63 (85%)	
Positive	21 (50%)	11 (15%)	
Liver metastases			0.51
Negative	30 (71%)	57 (77%)	
Positive	12 (29%)	17 (23%)	
Lung metastases			0.17
Negative	33 (79%)	66 (89%)	
Positive	9 (21%)	8 (11%)	
Differentiation			**0.02**
Differentiated	32 (76%)	69 (93%)	
Undifferentiated	10 (24%)	5 (7%)	
AJCC stage			**0.02**
I	0 (0%)	1 (1%)	
II	9 (21%)	31 (42%)	
III	11 (26%)	23 (31%)	
IV	22 (52%)	19 (26%)	

Bold number in the *P* value: the variables were considered to have statistical significance as *P* value ≤ 0.05.

SD, Standard deviation; CEA, Serum carcinoembryonic antigen; AJCC, American Joint Committee on Cancer, Differentiated, Well-differentiated and moderately differentiated; Undifferentiated, Poorly differentiated, undifferentiated.

*Comparison of validation cohort by χ^2^ test or unpaired t-test.

### CILP2 knockdown decreased the invasive and metastatic capacities of HCT116 cells

**Figure 4:**
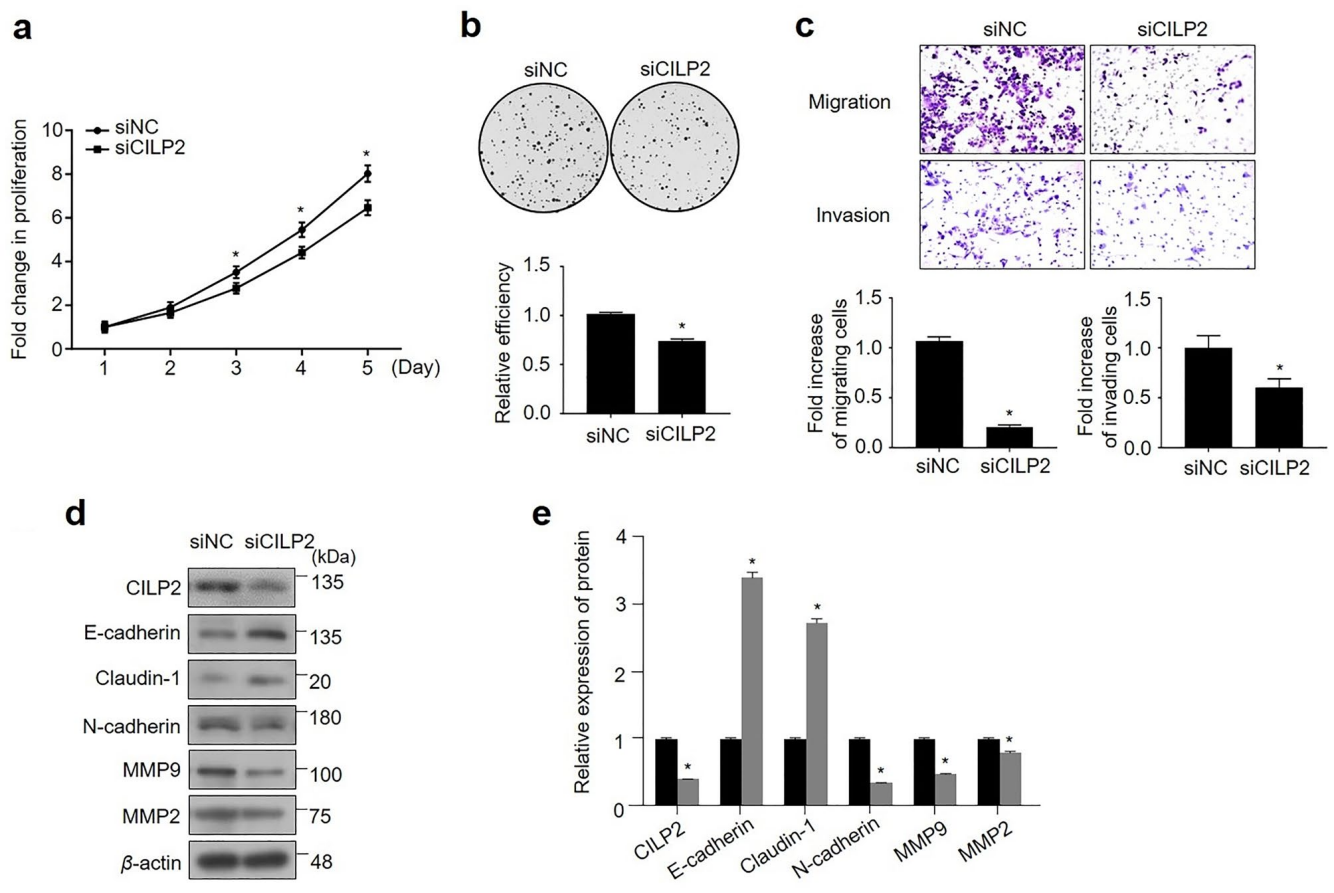
The small interfering RNA-mediated knockdown of CILP2 inhibiting the proliferation, colony formation, migration, invasion, and adhesion of HCT116 cells in vitro. (**a**) The knockdown of CILP2 decreased cell proliferation. (**b**) The knockdown of CILP2 decreased colony formation. (**c**) The knockdown of CILP2 significantly decreased cell migration and invasion. (**d**) The knockdown of CILP2 inhibited epithelial-mesenchymal transition markers and transcription factors in HCT116 cells. (**e**) The relative expression of each protein is determined as a ratio to β-actin. Each experiment is repeated at least three times. All data are expressed as the mean ± standard deviation; *P < 0.05.

To investigate whether CILP2 promotes PMCRC, cell-based functional assays, including proliferation, colony formation, invasion, and migration, were conducted. HCT116 cells were chosen for CILP2 loss-of-function experiments because of their highest mRNA expression of the gene among the five CRC cell lines (Fig. S2). We assessed CILP2 siRNA transfection efficiency at 72 h using RT-PCR and Western blotting (Fig. S3). Here, siCILP2 #3 exhibited the best transfection effects. Thus, we chose siCILP2 #3 for further experiments. The proliferation of CILP2-downregulated HCT116 cells decreased significantly in a time-dependent manner from days 3 to 5 compared with that of the control (Fig. 4a). The colony-forming assay displayed reduced colony formation by HCT116 cells with CILP2 downregulation (Fig. 4b). The transwell migration and Matrigel invasion assay results indicated that the migratory and invasive abilities of the cells significantly decreased following CILP2 knockdown compared to siNC cells (Fig. 4c). Subsequently, epithelial-mesenchymal transition (EMT)-related molecules were analysed using Western blotting after transfection with siCILP2. Additionally, EMT-related molecules contribute to cancer progression and metastases^21^. The results of this experiment demonstrated increased E-cadherin and Claudin-1 levels and decreased N-cadherin, MMP9, and MMP2 levels in the CILP2-knockdown HCT116 cells (Fig. 4d,e).

### CILP2 knockdown decreases the metastatic potential of CRC cells in vivo

**Figure 5:**
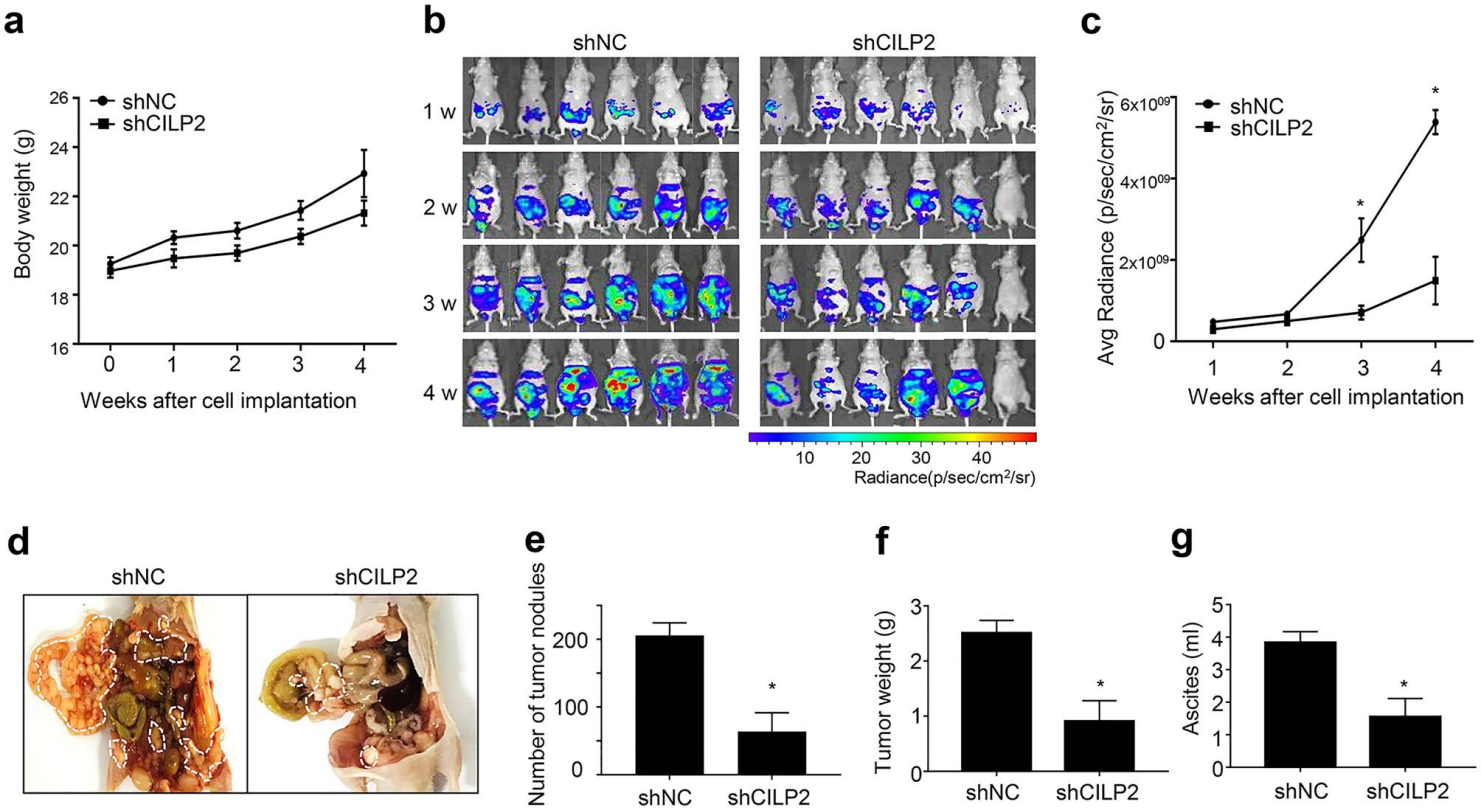
The downregulation of CILP2 decreases the potential metastases of colorectal cancer in vivo. (**a**) Changes in body weights in the shNC and shCILP2 groups. (**b**) In vivo images at 1, 2, 3, and 4 weeks after tumour dissemination (n = 6 per group). (**c**) Quantification of the in vivo imaging is displayed in the graph. (**d**) Representative images of the appearance of peritoneal metastatic nodules in the nude mice treated with an intraperitoneal injection. Numbers of peritoneal tumour nodules (**e**) total weight of the peritoneal tumour nodules (**f**), and total ascites fluid amounts (**g**) were measured after sacrificing mice.

To assess CILP2’s role in promoting PMCRC in vivo, stable CILP2 knockdown lines were established from HCT116-Luc parental cells and injected into nude mice to generate a mouse model of this disease. The transfection efficiency of CILP2 shRNA was measured using RT-PCR and Western blotting (Fig. S4). Here, shCILP2 D exhibited the best transfection effects. Thus, we chose shCILP2 D for further experiments. ShCILP2-transfected HCT116-Luc cells were injected into the peritoneal cavity of the nude mice to generate mouse models of PMCRC, which were used to evaluate the effect of CILP2 on peritoneal implantation. The progression of the tumours derived from the parental cells was detected using IVIS at 1, 2, 3, and 4 weeks after injection (Fig. 5). The tumours of the mice injected with HCR116-Luc-shCILP2 cells were significantly lower in weight compared with the mice injected with the negative control of shRNA (shNC) (Fig. 5a). Luciferase activity was also significantly reduced in mice inoculated with shCILP2 cells compared with those inoculated using the shNC cells (Fig. 5b). Quantitative analysis revealed reduced tumour growth in the HCR116-Luc-shCILP2 mice compared to the controls. The time course of tumour growth was determined through the quantification of photon counts (Fig. 5c). The number of peritoneal metastatic nodules was significantly reduced in the HCT116-Luc-shCILP2 group compared to the HCT116-Luc-shNC group (Fig. 5d,e). Furthermore, a significant difference was observed in the tumour weights between the shNC and shCILP2 groups (Fig. 5f). Finally, in HCT116-shCILP2 mice, ascites formed in the peritoneal cavities were remarkably reduced in number (Fig. 5g).

## Discussion

Significant treatment challenges are posed by PM owing to multiple reasons. Early detection of PM through imaging and achieving a complete resection that includes micrometastases are difficult^22^. Additionally, CRC cells exhibit poor sensitivity to radiation, and drug delivery to the peritoneal surface is limited^23–25^. Therefore, a critical need exists for new predictive biomarkers specific to PM in CRC to address early treatment complexities. The identification of new molecular targets is also likely to contribute to the evaluation and timely delivery of effective cytotoxic therapy.

In this study, we aimed to identify molecules responsible for mediating the onset of PM in CRC. We performed transcriptomic profiling analysis and filtered out 1,422 genes that were differentially expressed in the PM group compared with the group without metastases. To identify genes specifically associated with PM, we excluded genes that displayed significant expression differences between the groups with liver or lung metastases compared with the groups without metastases. We utilised both RNA-seq data from our prior study and the current analysis. We used the primary tumours from CRC patients for RNA-seq. Some studies report high concordance between peritoneal lesions and their primary tumours^1,26,27^. Lenos et al. demonstrated that peritoneal lesions seemed to have much more similarity to their primary tumour compared to other metastases, and these lesions seemed to retain both clonal heterogeneity and transcriptional profile^1^. This evidence has displayed that the activation of the metastatic potential is already encoded in the primary tumour at early stages rather than subsequently acquired by clonal expansions of an ancestral fraction of tumour cells with metastatic potential.

Subsequently, the 12 genes were selected by more stringent criteria and validated in an independent cohort using real-time RT-PCR. Among these genes, CILP2 and KRT6A exhibited high expression in the PM group compared to the non-PM group, consistent with the RNA-seq results. The KRT6A gene displayed a significantly consistent expression pattern in our cohorts. However, the high expression of KRT6A did not appear to contribute to tumorigenesis and metastases, as the high-expression group of CRC had a better prognosis than the low-expression group of CRC in the TCGA cohort. By contrast, CILP2 had significantly consistent expression in our cohorts, and the high-expression group of CRC had a worse prognosis than the low-expression group of CRC.

The CILP2 gene encodes cartilage intermediate layer protein-like protein 2, which is a non-collagenous protein in human articular cartilage. Most previous studies on CILP2 have reported a correlation with lipid metabolism^28–30^. Notably, the molecular mechanism by which CILP2 affects lipid metabolism remains unclear. Several studies have reported the involvement of CILP2 in cancer progression. An expression quantitative trait locus for the CILP2 gene, rs8103992, was statistically significantly associated with adult height attainment and osteosarcoma risk after adjustment for multiple comparisons^31^. Huang et al. reported that this protein is associated with advanced-stage lesions and can play a role as an independent predictor of poor survival in CRC^32^. Wang et al. reported that elevated CILP2 expression is associated with adverse CRC clinical features and immune cells, making it a potentially unfavourable biomarker for CRC survival^33^. To the best of our knowledge, this study is the first to demonstrate that CILP2 expression is associated with the development of PM from CRC.

To assess whether the CILP2 levels in CRC tissues served as a diagnostic and predictive marker for PM in CRC, we determined an optimal cut-off value for this purpose by analysing CILP2 mRNA expression in the validation cohort. We identified a close association in the validation cohort between CILP2 levels and PM. CILP2 expression levels in primary CRC tissues were significantly higher in patients with PM compared with those without, suggesting its potential for identifying those at risk for PM. Moreover, OS times were short in patients with CRC who exhibited higher CILP2 expression in our validation cohort, consistent with the TCGA cohort. Elevated CILP2 expression was significantly associated with the presence of PM and risk factors such as differentiation and tumour stage. These findings support the hypothesis that CILP2 expression reflects the potential of primary tumour cells to metastases to the peritoneal cavity.

The PM activation process broadly comprises the loss of intracellular adhesion and polarity, leading to increased migratory and invasive properties of the tumour cells^34–36^. Our functional assays with artificial modulation of CILP2 expression suggested that CILP2 expression in the HCT116 cell line promotes malignant phenotypes, such as colony formation, migration, and invasion into the extracellular matrix. EMT activation, a well-described mechanism leading to major morphogenetic events, endows cells with migratory and invasive capabilities^37^. Down-regulation of E-cadherin and Claudin-1 expression and up-regulation of N-cadherin expression are the main characteristics of the EMT process^38^. Our data demonstrated that silencing CILP2 inactivated the EMT process in HCR116 cells. Extracellular matrix degradation plays an important role in tumour invasion and metastases, which is mainly mediated by MMP2 and MMP9^39^. Our data also demonstrated that silencing CILP2 downregulated MMP2 and MMP9 in HCT116 cells. These findings support the hypothesis that CILP2 is an oncoprotein that contributes to the development of PM. Furthermore, our in vivo studies exhibited that CILP2 knockdown in CRC cells significantly downregulated tumour nodules, tumour weight, and ascites, indicating a decreased ability for cancer cell dissemination and growth both in vitro and in vivo. These findings support the conclusion that CILP2 serves as a biomarker and a therapeutic target for PM in CRC.

This study had two notable limitations. First, the mechanism of overexpressed CILP2 in CRC patients with PM remains unknown. Further studies are required to elucidate the pathways and proteins that interact with CILP2 to understand its biological functions in CRC. Second, an orthotropic model of engraftment was not employed in our analyses; therefore, evaluating the influence of CILP2 on the detachment of cancer cells from the primary tumour was not possible. However, we utilised an intraperitoneal injection model to demonstrate the capacity of disseminated cells to survive, adhere to the peritoneal lining, and proliferate in this non-native location. Some previous studies displayed this model is well-suited for unravelling the pathophysiological mechanisms of PM and investigating potential novel drug targets and other therapeutic strategies^40,41^.

In summary, the results of our current study suggest the potential value of CILP2 as a promising biomarker for predicting PM in CRC and are likely to contribute to the identification of novel treatment targets for these patients.

### Supplementary Information

Supplementary Information 1.

Supplementary Information 2.

Supplementary Information 3.

Supplementary Information 4.

Supplementary Information 5.

Supplementary Information 6.

Supplementary Information 7.

Supplementary Information 8.

Supplementary Information 9.

## Data Availability

The data sets used in this study are available at the NCBI' Gene Expression Omnibus (GSE225182). The raw data was deposited in Korean Nucleotide Archive (KoNA, https://kobic.re.kr/kona) with the accession ID, PRJKA230588.
