# Supplementary figures and images for: CILP2 is a potential biomarker for the prediction and therapeutic target of peritoneal metastases in colorectal cancer
Source: Sci Rep. 2024 May 31;14:12487. doi: 10.1038/s41598-024-63366-4 (PMC11139887; doi:10.1038/s41598-024-63366-4)

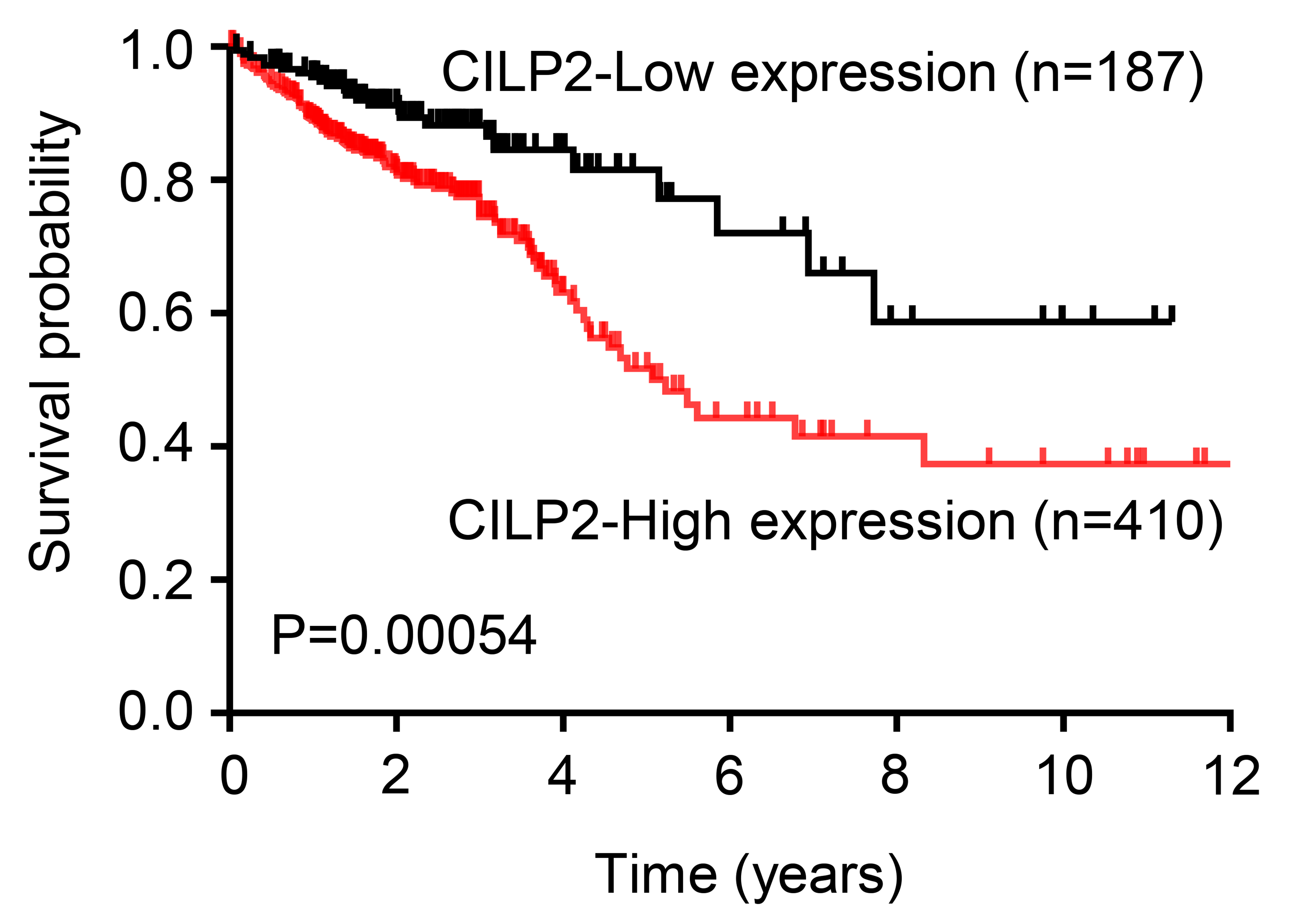

Supplement: Supplementary file 1 — Supplementary Information 1. [file 41598_2024_63366_MOESM1_ESM.tif]

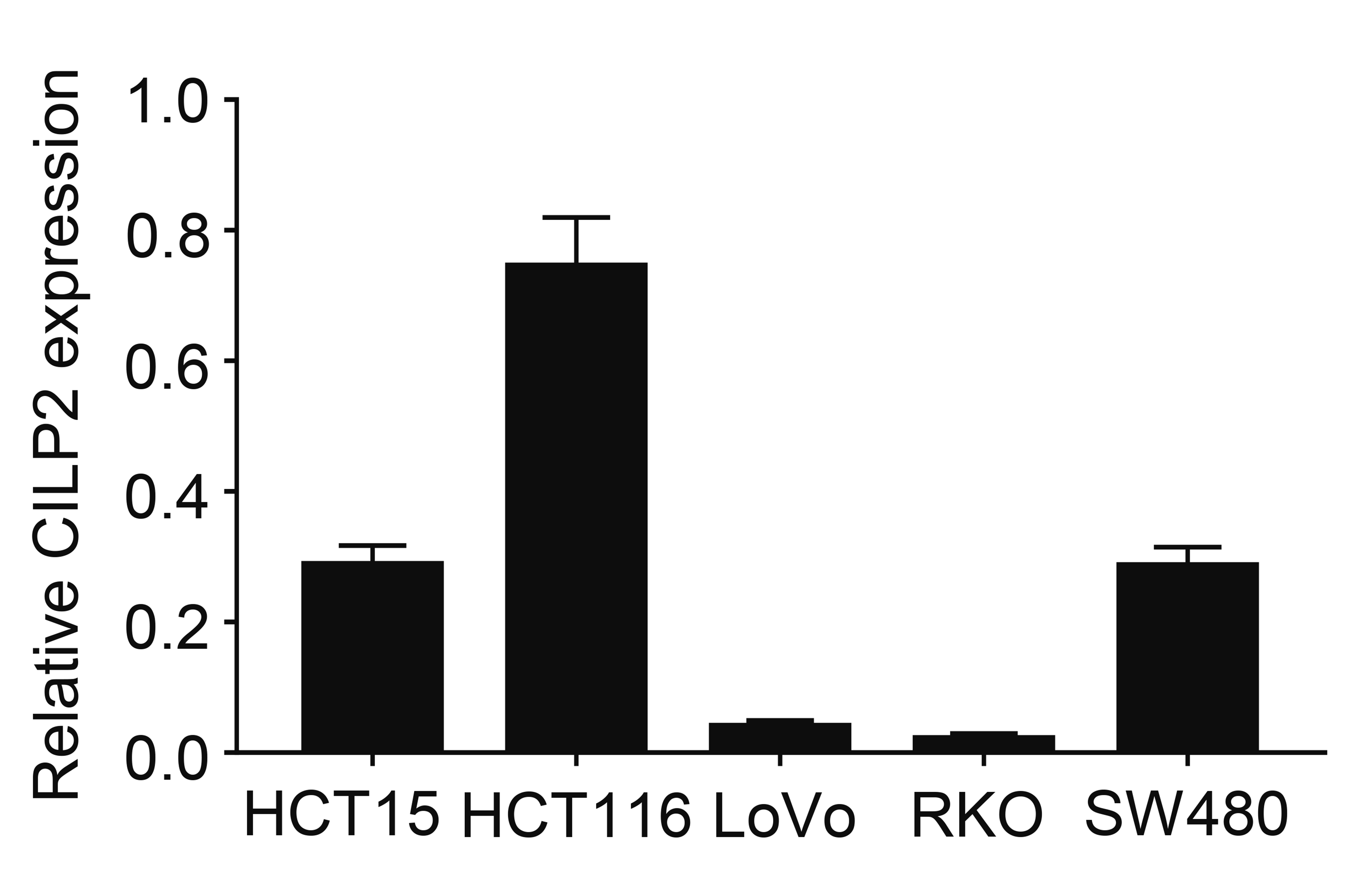

Supplement: Supplementary file 2 — Supplementary Information 2. [file 41598_2024_63366_MOESM2_ESM.tif]

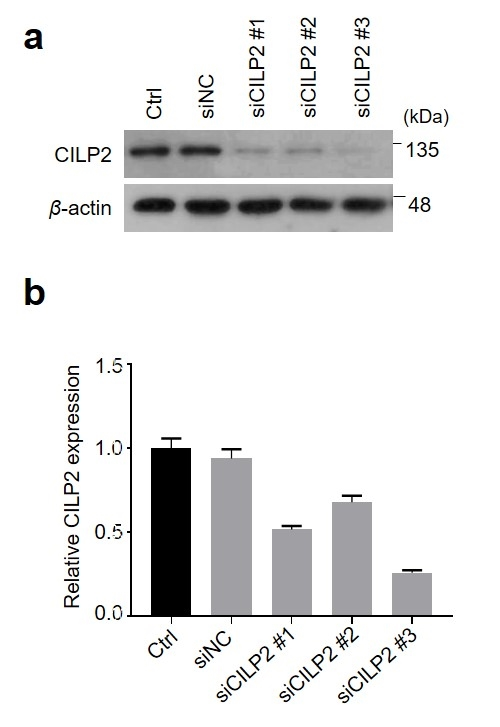

Supplement: Supplementary file 3 — Supplementary Information 3. [file 41598_2024_63366_MOESM3_ESM.tif]

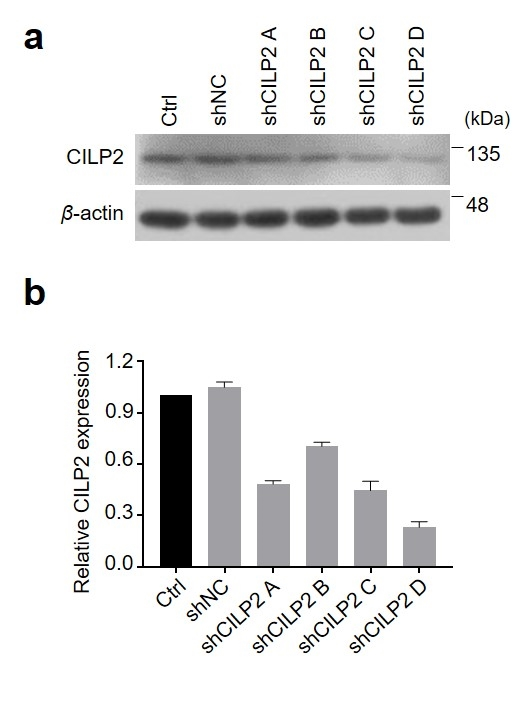

Supplement: Supplementary file 4 — Supplementary Information 4. [file 41598_2024_63366_MOESM4_ESM.tif]

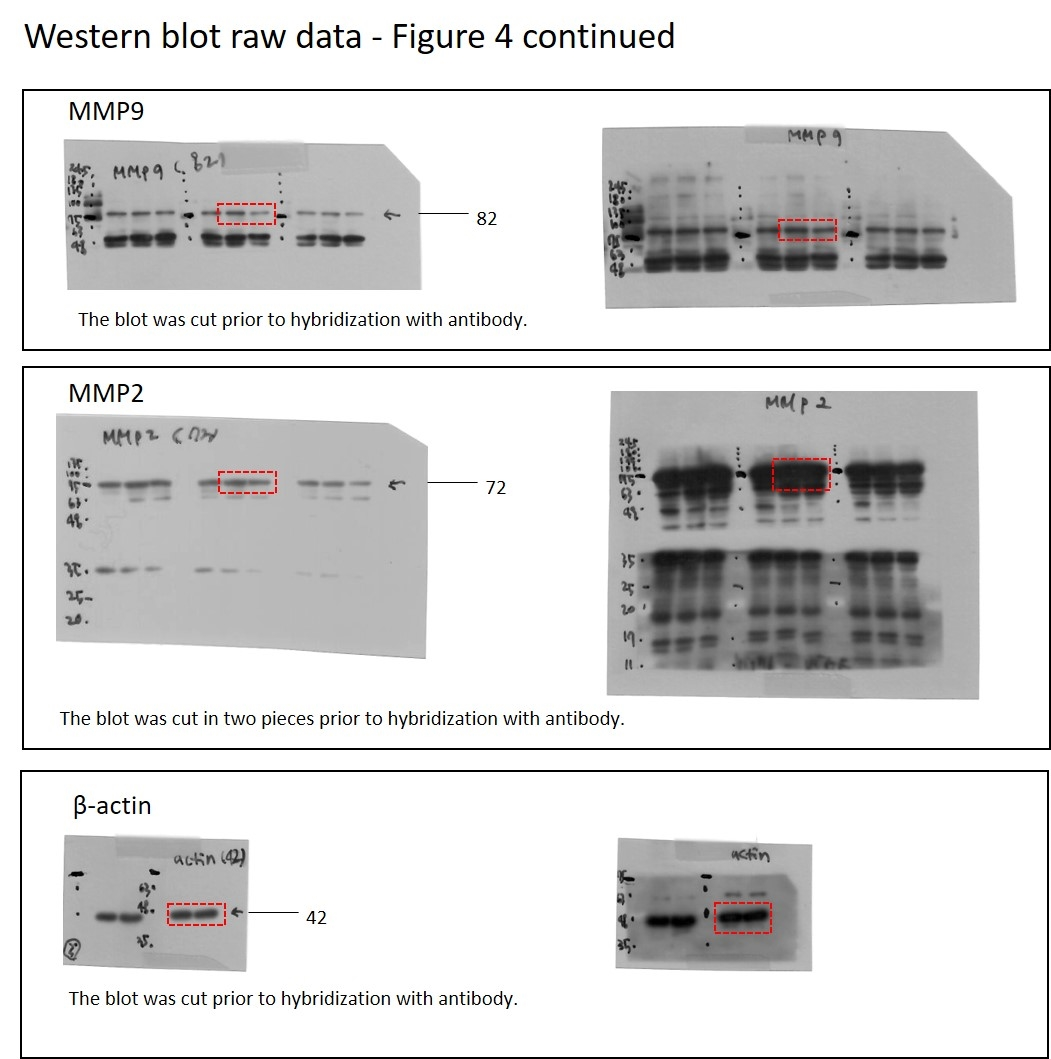

Supplement: Supplementary file 6 — Supplementary Information 6. [file 41598_2024_63366_MOESM6_ESM.tif]

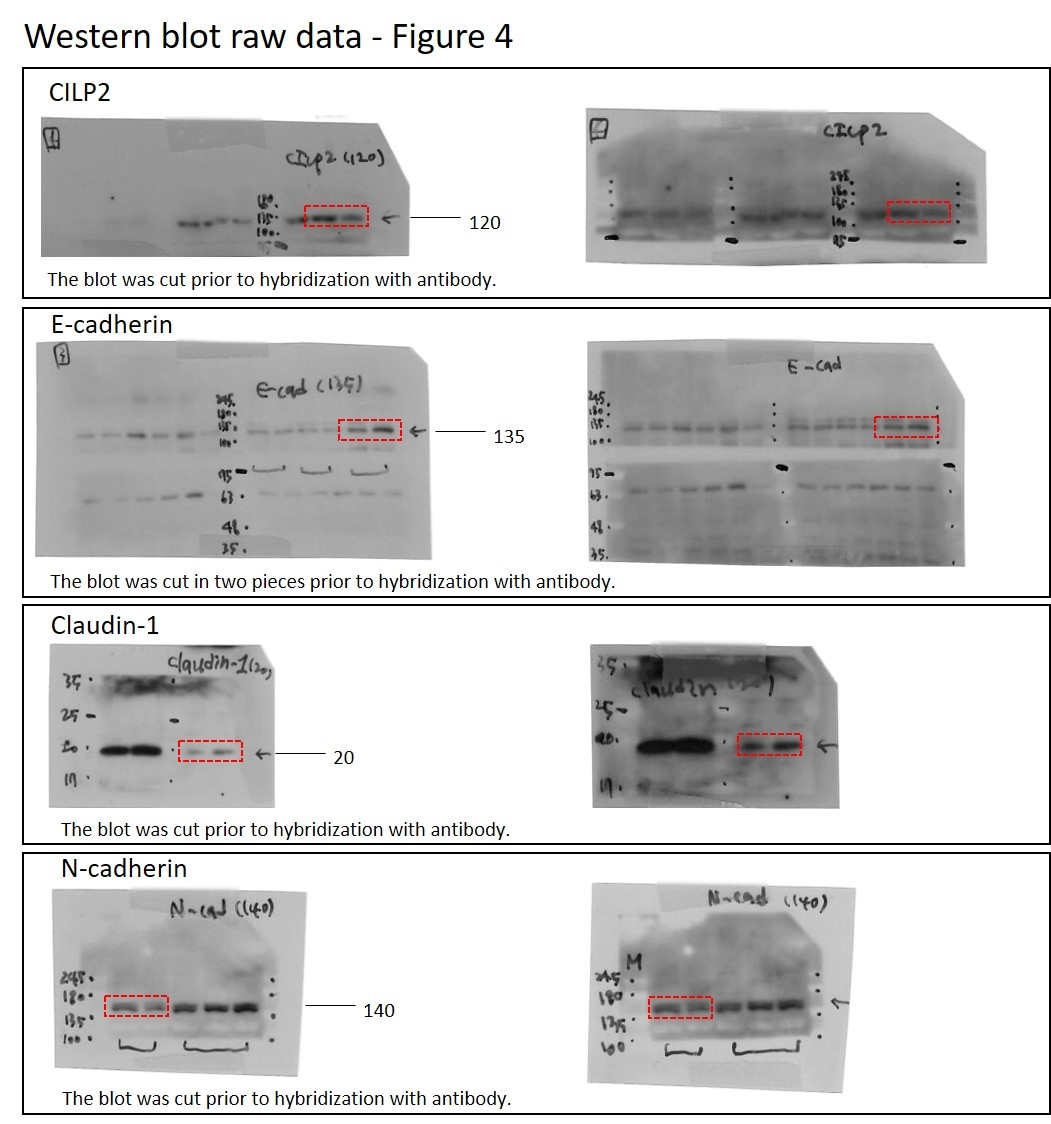

Supplement: Supplementary file 7 — Supplementary Information 7. [file 41598_2024_63366_MOESM7_ESM.tif]

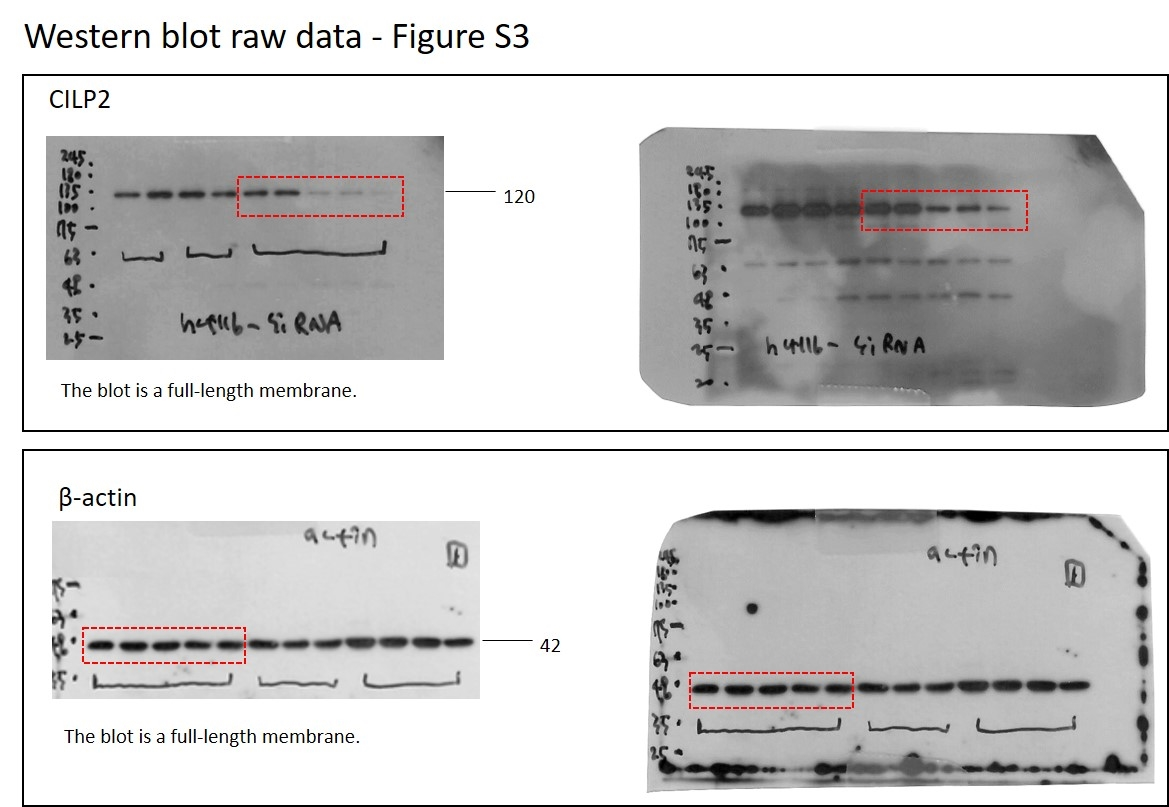

Supplement: Supplementary file 8 — Supplementary Information 8. [file 41598_2024_63366_MOESM8_ESM.tif]

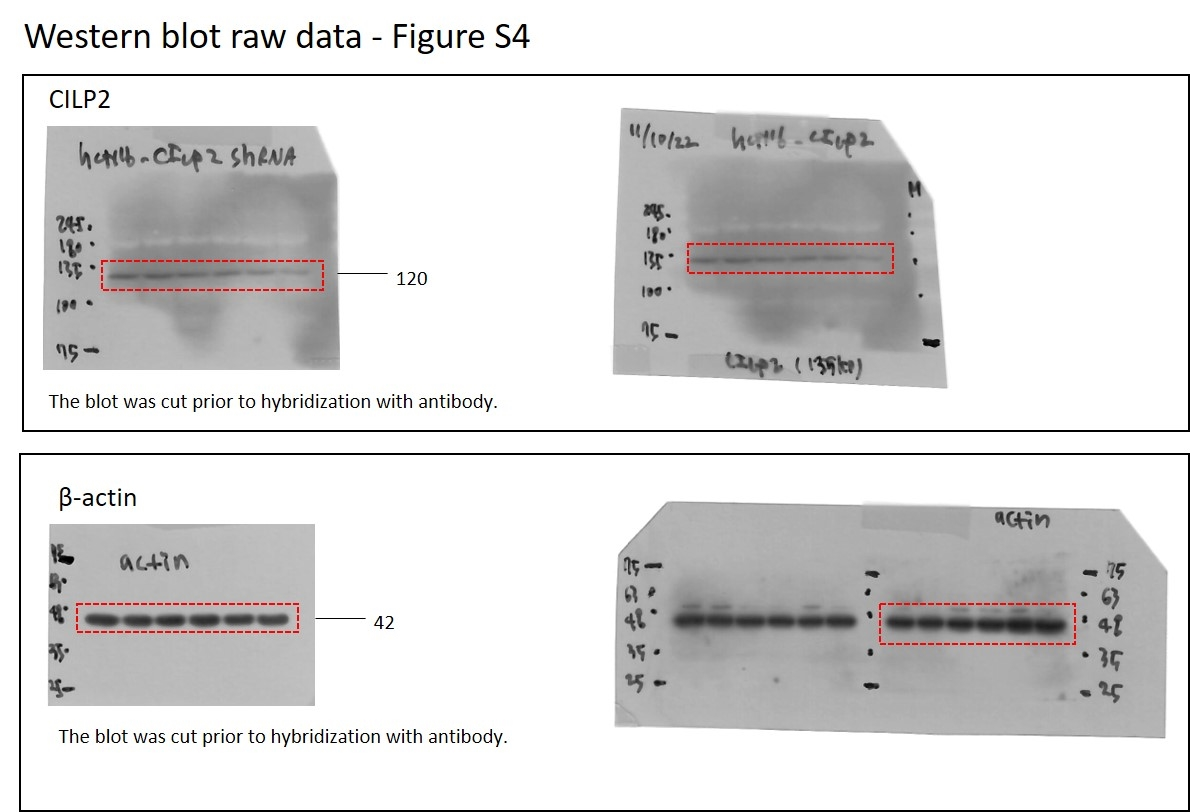

Supplement: Supplementary file 9 — Supplementary Information 9. [file 41598_2024_63366_MOESM9_ESM.tif]
